# 4’-O-Methylbroussochalcone B as a novel tubulin polymerization inhibitor suppressed the proliferation and migration of acute myeloid leukaemia cells

**DOI:** 10.1186/s12885-020-07759-4

**Published:** 2021-01-22

**Authors:** Ziying Liu, Changshui Wang, Yali Wang, Lei Wang, Yueyuan Zhang, Genquan Yan

**Affiliations:** 1 Department of pediatrics, Affiliated Hospital of Jining Medical University, Jining Medical University, Jining, China; 2 Department of Clinical & Translational Medicine, Jining Life Science Center, Jining, China; 3 Department of pharmacy, Shandong Provincial Hospital Affiliated to Shandong First Medical University, Jinan, China

**Keywords:** 4′-O-Methylbroussochalcone B, Acute myeloid leukaemia, Migration, Colchicine, Tubulin polymerization inhibitor

## Abstract

**Background:**

Recent years, survival rates of human with high-risk acute myeloid leukaemia (AML) have not raised substantially. This research aimed to investigate the role of 4′-O-Methylbroussochalcone B, for the treatment of human AML.

**Methods:**

Firstly, we evaluated the effects of six chalcones on AML cells activity by MTT assay. Immunofluorescence staining, tubulin polymerization assay and N,N′-ethylenebis (iodoacetamide) (EBI) competition assay were performed on ML-2 cells. Transwell and apoptosis assay were also utilized in ML-2 cells and OCI-AML5 cells. The expressions of migration-related proteins, apoptosis-related proteins and Wnt/β-catenin pathway were detected by Western Blot.

**Results:**

The results found six chalcones exhibited the anti-proliferative activity against different AML cell lines. Based on the results of immunofluorescence staining, tubulin polymerization assay and EBI competition assay, 4′-O-Methylbroussochalcone B was discovered to be a novel colchicine site tubulin polymerization inhibitor. 4′-O-Methylbroussochalcone B could induce apoptosis, inhibit proliferation and migration of ML-2 cells and OCI-AML5 cells. The cells were arrested in the G2-M phase by the treatment of 4′-O-Methylbroussochalcone B. In addition, 4′-O-Methylbroussochalcone B regulated MAPK and Wnt/β-catenin pathways in AML cells.

**Conclusion:**

4′-O-Methylbroussochalcone B might inhibit proliferation and migration of the AML cells by MAPK and Wnt/β-catenin pathways as a tubulin polymerization inhibitor. It is promising for 4′-O-Methylbroussochalcone B to become a new drug to treat AML.

**Supplementary Information:**

The online version contains supplementary material available at 10.1186/s12885-020-07759-4.

## Background

Human acute myeloid leukaemia (AML) as a hematologic malignancy is characterized by the accumulation of genetic changes in hemopoietic progenitor cells that regulate mechanisms of differentiation, proliferation and self-renewal [1, 2]. The mortality rates of human cancers in the United States have decreased by more than 50% from 1975 to 2010, and the 5-year survival rate for AML children has increased to 68% [3–5]. Although the therapeutic approaches of human AML have gradually improved, survival outcomes have not improved substantially and approximately more than half of children AMLs suffered from disease recurrence [6]. Therefore, it is very necessary to develop novel anticancer drugs to treat human AML.

Microtubules as crucial elements of the cytoskeleton play significant roles in a wide range of cellular functions, such as maintenance of cell shape, division, motility, and intracellular vesicle transport [7]. Recently, microtubules have been represented as an attractive target in anticancer drug discovery [8, 9]. Based on their effects on microtubule dynamics, microtubule-targeting agents are generally classified into two categories: microtubule-destabilizing and microtubule-stabilizing agents [10, 11]. Importantly, these microtubule-targeting agents exhibited a powerful cytotoxicity against a large number of cancer cell lines, including human AML cells [12, 13].

Natural products from medicinal plants have long been a rich resource for identifying novel anticancer agents with relatively few side effects [14]. The seeds of *Cullen corylifolium* (L.) Medik. (syn. *Psoralea corylifolia* L.) (Leguminosae) have been used traditionally for treatment of vitiligo, gynaecological bleeding, and skin diseases [15].

Its seeds were reported to contain chalcones (4′-O-Methylbroussochalcone B, Broussochalcone B, Isobavachalcone, Bavachromene, Isobavachromene, and Dorsmanin A) as potentially antitumor agents [15, 16]. However, the anticancer actions of 4′-O-Methylbroussochalcone B against AML remain unclear. In this study, we detected the antiproliferative activity of 4′-O-Methylbroussochalcone B, Broussochalcone B, Isobavachalcone, Bavachromene, Isobavachromene, and Dorsmanin A (**Fig. S****1**) against different AML cell lines. Importantly, 4′-O-Methylbroussochalcone B was identified as a potent tubulin polymerization inhibitor that inhibited proliferation and epithelial-mesenchymal transition in AML cells.

## Methods

### Materials

Chalcones including 4′-O-Methylbroussochalcone B (catalog no. ALB-RS-3963, ALB Technology Limited, USA), Broussochalcone B (catalog no. HY-N0231, MedChemexpress LLC, USA), Isobavachalcone (catalog no. CFN98593, ChemFaces, Wuhan, China), Bavachromene (catalog no. CFN92219, ChemFaces, Wuhan, China), Isobavachromene (catalog no. CFN92220, ChemFaces, Wuhan, China), and Dorsmanin A (catalog no. CFN96193, ChemFaces, Wuhan, China), from *Cullen corylifolium* were used in this study. GDM-1 cells (catalog no. BNCC307472), ML-2 cells (catalog no. BNCC353358) and CESS cells (catalog no. BNCC254816) were purchased from BeiNa Culture Collection (Shanghai, China) in May. 2019. OCI-AML5 (catalog no. ACC 247) cells was obtained from DSMZ in May. 2019. They were cultured in 89% RPMI medium, 10% FBS, 1% streptomycin and penicillin, respectively, all of which were obtained from Life Technologies (California, USA). 3-(4,5-dimethylthiazol-2-yl)-2,5-diphenyltetrazolium bromide (MTT) was purchased from Sigma-Aldrich (St Louis, MO, USA). Annexin V-FITC /PI (catalog no. KGA108) was purchased from KeyGen Biotech (Jiangsu, China).

### Cell culture and MTT assay

Cells were grown in a 5% CO_2_ incubator at 37 °C. We used trypsin to digest cells in logarithmic growth phase. Then, we plated the cells in 96-well plates at 5000 cells *per* well evenly. After cell adherence, chalcones were added in the 96-well plates for 48 h or 72 h. We added MTT reagent to each well and incubated for 2 h in the incubator. Finally, the absorbance value was measured at 490 nm after treatment with DMSO.

### Immunofluorescence staining

ML-2 cells were seeded into 6-well plates and then treated with 4′-O-Methylbroussochalcone B for 24 h. ML-2 cells were washed with phosphate buffer solution (PBS), and then fixed in 4% paraformaldehyde. After blocking for 0.5 h in 5% goat serum albumin, ML-2 cells were incubated with anti-β-tubulin for 2 h. Then, ML-2 cells were washed by PBS and labeled by 4, 6-diamidino-2-phenylindole.

### Tubulin polymerization assay in vitro

An amount of 2 mg/mL tubulin was resuspended in PEM buffer, consisted of 80 mM PIPES (pH 6.9), 2 mM MgCl_2_, 0.5 mM EGTA, and 15% glycerol. 4′-O-Methylbroussochalcone B (1 μM, 3 μM and 6 μM) was preincubated on ice. The reaction was monitored by a spectrophotometer in absorbance at 340 nm at 37 °C.

### EBI competition assay

ML-2 cells were seeded in 6-well plates for 24 h. 4′-O-Methylbroussochalcone B and colchicine treated them for 2 h respectively. Then, EBI were added. After 90 min, ML-2 cells were harvested to do Western Blot analysis. The signals were then visualized using chemiluminescent substrate (Zhengzhou research company, Zhengzhou, China).

### Cell cycle assay

The samples of fixed cells were stained with 50 μg/ml Propidium iodide (PI, Sigma) in the dark for 30 min at room temperature, following the manufacturer’s protocol. Then the samples were tested by flow cytometer (Becton, Dickinson and Company, NJ). Data were analyzed via CellQuest software.

### Transwell assay

The cells (100 μl, 1 × 10^6^ cells/ml) were suspended in the medium which contained 0.1% FBS and plated to the upper chamber of the transwell. The lower wells were incubated with the addition of the medium (600 μl per well), which included CCL25 (100 ng/ml; Cedarlane Laboratories, Burlington, Canada) and 0.1% FBS. The cells were hatched for 24 h at 37 °C with 5% CO_2_. Finally, the cells in the lower chamber were counted under a light microscope.

### Apoptosis assay

ML-2 cells and OCI-AML5 cells were seeded in 6-well culture plates and treated with certain concentration of 4′-O-Methylbroussochalcone B for 48 h. Cells were harvested and suspended in binding buffer containing Annexin V-FITC (0.5 mg/mL) and PI (0.5 mg/mL) then. After that, samples were incubated for 25 min in dark and analyzed with flow cytometry (Becton, Dickinson and Company, NJ).

### Western blotting analysis

The proteins in ML-2 cells and OCI-AML5 cells lysates were resolved by polyacrylamide gel electrophoresis and transferred to polyvinylidene fluoride membrane. Subsequently, the membranes were incubated with the primary antibodies overnight at 4 °C. Goat anti-rabbit IgG-HRP (catalog no. SA00001–2, proteintech, USA) served as the second antibodies. The signals were then visualized using chemiluminescent substrate. We detected the intensity of protein bands by the Quantity One software (Bio-rad, Richmond, California, USA).

### Statistical analysis

Data are shown as the means ± standard error of the mean (SEM) from at least three independent experiments. Analysis of variance (ANOVA) together with Neumann-Keul’s multiple comparison test or Kolmogorov-Smirnov test were performed to obtain statistical information.

## Results

### Antiproliferative effects of natural chalcones from Cullen corylifolium

**Fig. 1:**
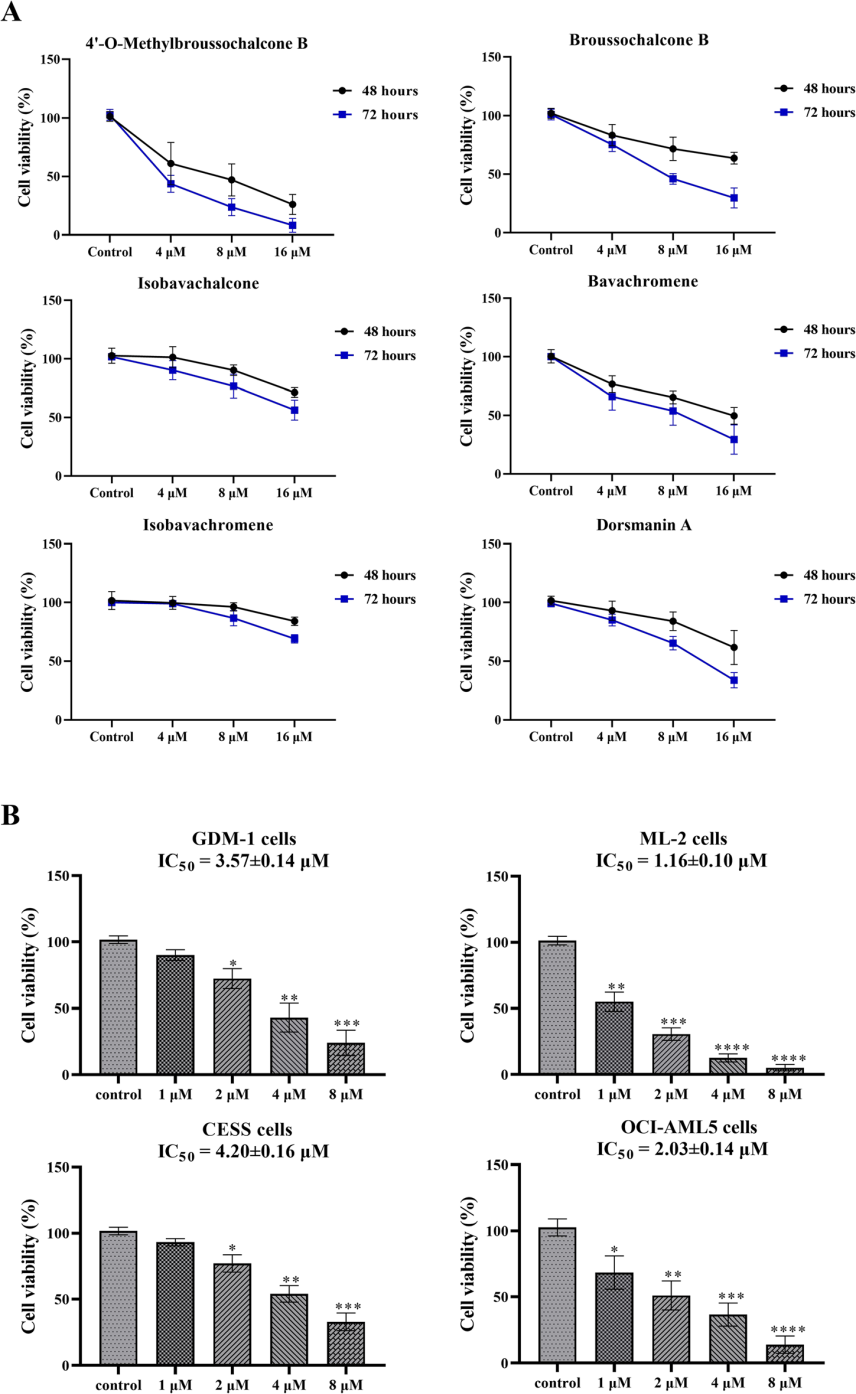
Antiproliferative effects of natural chalcones from *Cullen corylifolium* against AML line cells. **a** Antiproliferative effects of natural chalcones from *Cullen corylifolium* against GDM-1 cells detected by MTT; **b** Antiproliferative effects of 4′-O-Methylbroussochalcone B at different concentrations (control, 1 μM, 2 μM, 4 μM and 8 μM) against GDM-1 cells, ML-2 cells, CESS cells, OCI-AML5 cells for 72 h detected by MTT. The data were presented as the mean ± SEM *P < 0.05, **P < 0.01, ***P < 0.001 and ****P < 0.0001 vs the control

Six chalcones (4′-O-Methylbroussochalcone B, Broussochalcone B, Isobavachalcone, Bavachromene, Isobavachromene, and Dorsmanin A) from *Cullen corylifolium* were evaluated for their antiproliferative effects against GDM-1 cells using the MTT (thiazolyl blue tetrazolium bromide) assay. The antiproliferative results were shown in Fig. 1a. After treatment of GDM-1 cells with 16 μM 4′-O-Methylbroussochalcone B, Broussochalcone B, Isobavachalcone, Bavachromene, Isobavachromene, and Dorsmanin A for 72 h, the cell proliferation rates were 8.14, 29.67, 57.33, 29.40, 69.00 and 36.67%, respectively. Based on the above results, 4′-O-Methylbroussochalcone B displayed the strongest antiproliferative effects among all six chalcones.

### 4′-O-Methylbroussochalcone B potently inhibited the proliferation against different AML cell lines

Then, we further assessed the antiproliferative actions of 4′-O-Methylbroussochalcone B against different AML cell lines. As depicted in Fig. 1b, 4‘-O-Methylbroussochalcone B exhibited the potently antiproliferative effects against GDM-1 cells, ML-2 cells, CESS cells and OCI-AML5 cells with IC_50_ values of 3.57 μM, 1.16 μM, 4.20 μM and 2.03 μM, respectively. It was observed that 4’-O-Methylbroussochalcone B showed the best antiproliferative effect of on ML-2 cells, followed by OCI-AML5 cells.

### 4′-O-Methylbroussochalcone B inhibited tubulin polymerization in vitro

To investigate whether the activities of the natural chalcones from *Cullen corylifolium* were related to the interactions with microtubule systems, all six chalcones were measured the inhibition of tubulin polymerization. Among six chalcones, 4′-O-Methylbroussochalcone B showed a concentration-dependent inhibition of tubulin polymerization with calculated IC_50_ values of 2.86 μM, which displayed the best inhibition. (**Table S****1**).

The tubulin assembly assay measured the extent of assembly of 2 mg/mL tubulin after 60 min at 37 °C. Data are presented as mean from three independent experiments.

### 4′-O-Methylbroussochalcone B inhibited tubulin polymerization in ML-2 cells

**Fig. 2:**
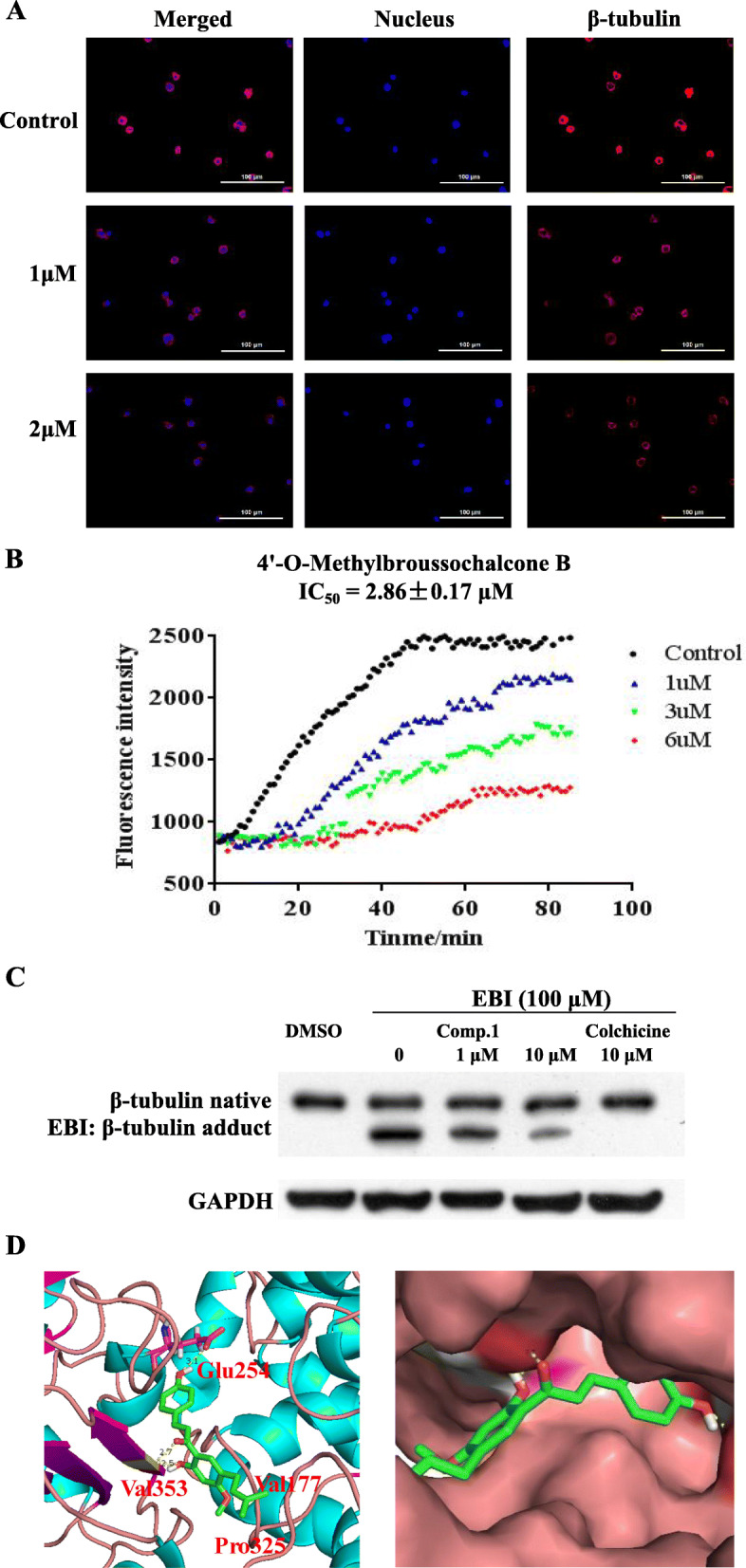
4′-O-Methylbroussochalcone B as a novel tubulin polymerization inhibitor directly bind to the cochicine binding site of β-tubulin. **a** Immunofluorescence staining of β-tubulin inhibited by 4′-O-Methylbroussochalcone B in ML-2 cells; **b** Tubulin polymerization assay was admeasured for the inhibitory activity of 4 ‘-O-Methylbroussochalcone B on tubulin polymerization in vitro; **c** EBI assay combined with Western Blot analysis was used to in ML-2 cells, full-length blots/gels are presented in Supplementary Fig. S2a; **d** Molecular modeling study (PDB code 1SA0)

To explore the effects of 4′-O-Methylbroussochalcone B on tubulin polymerization in ML-2 cells, we performed ML-2 cells treating with 4′-O-Methylbroussochalcone B at different concentrations (control, 1 μM and 2 μM) to examine its effect on microtubules reorganization. As shown in Fig. 2a, the expression of β-tubulin was suppressed in a concentration-dependent manner, indicating that 4′-O-Methylbroussochalcone B might be a potential tubulin polymerization inhibitor.

### 4′-O-Methylbroussochalcone B inhibited tubulin polymerization by targeting the colchicine binding site

The inhibition of 4′-O-Methylbroussochalcone B on tubulin polymerization in vitro was evaluated in this work. As shown in Fig. 2b, with the elevated 4′-O-Methylbroussochalcone B (1 μM, 3 μM, 6 μM), the increased tendency of the fluorescence intensity was obviously slowed down.

In the EBI assay, preincubation of 4′-O-Methylbroussochalcone B at 10 μM prevented the formation of EBI: β-tubulin adduct which resulting in the decrease of the adduct band in comparison with EBI treatment and 1 μM 4′-O-Methylbroussochalcone B treatment (Fig. 2c). These results indicated that 4′-O-Methylbroussochalcone B directly binds to the colchicine binding site of β-tubulin.

The molecular docking study was carried out to elucidate the binding features of 4′-O-Methylbroussochalcone B with tubulin. Since the α-tubulin and β-tubulin heterodimers are complex with different ligands in certain flexibility at the colchicine site, we performed the docking studies of 4′-O-Methylbroussochalcone B with the representative crystal structure (PDB code: 1SA0). As shown in Fig. 2d, two hydroxyl groups attaching to the chalcone scaffold could form two hydrogen bonds with the residues Val353 and Glu254. The carbonyl group on 4′-O-Methylbroussochalcone B also formed a hydrogen bond with the residue Val353. The 2-methylpent-2-ene group attaching to the phenyl fragment formed the hydrophobic interactions with the residues of Val177 and Pro325. All these results indicated that 4′-O-Methylbroussochalcone B could target tubulin.

### ML-2 cells and OCI-AML5 cells were arrested in the G2-M phase by 4′-O-Methylbroussochalcone B

**Fig. 3:**
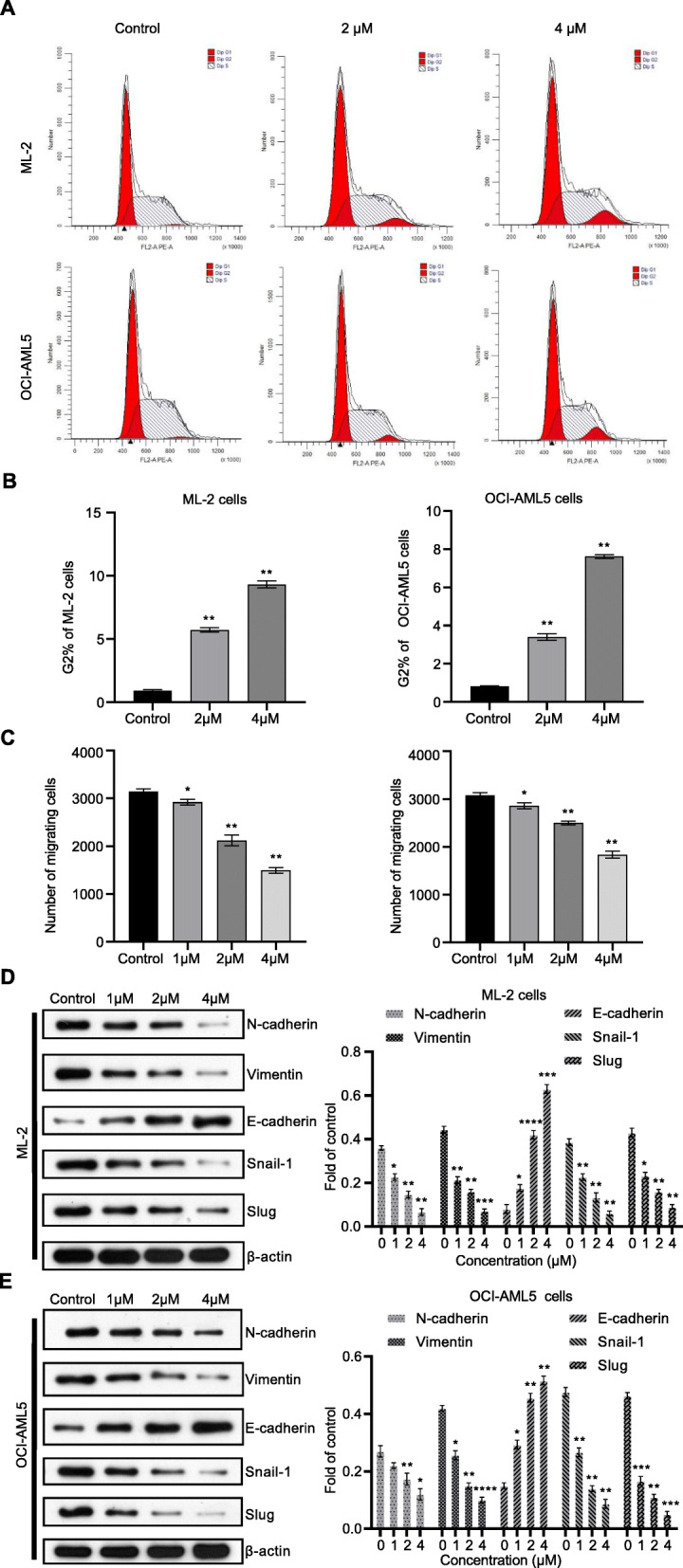
4′-O-Methylbroussochalcone B inhibited G2-M phase and migration in ML-2 cells and OCI-AML5 cells. **a**-**b** Cell cycle assay for ML-2 cells and OCI-AML5 cells at different concentrations of 4′-O-Methylbroussochalcone; **c** Transwell assay for ML-2 cells and OCI-AML5 cells at different concentrations of 4′-O-Methylbroussochalcone; **d** Western Blot measured the expression level of N-cadherin, Vimentin, E-cadherin, Snail-1, and Slug in ML-2 cells, full-length blots/gels are presented in Supplementary Fig. S2B; **e** Western Blot measured the expression level of N-cadherin, Vimentin, E-cadherin, Snail-1, and Slug in OCI-AML5 cells, full-length blots/gels are presented in Supplementary Fig. S2C. The data were presented as the mean ± SEM *P < 0.05, **P < 0.01, ***P < 0.001 and ****P < 0.0001, vs the control

Given that 4′-O-Methylbroussochalcone B could markedly suppressed the tubulin polymerization in ML-2 cells and OCI-AML5 cells, we further performed cell cycles assay to explore the role of 4′-O-Methylbroussochalcone B. The cells were incubated with different concentrations (0, 2 μM and 4 μM) of 4′-O-Methylbroussochalcone B for 48 h. Based on the results in Fig. 3a and b**,** for ML-2 cells and OCI-AML5 cells, the percentage of the cells in G2-M phase of 2 μM and 4 μM groups was dramatically increased respectively, compared with control group. All the results suggested that ML-2 cells and OCI-AML5 cells were arrested in the G2-M phase significantly by 4′-O-Methylbroussochalcone B in a concentration-dependent manner.

### 4′-O-Methylbroussochalcone B inhibited migration in ML-2 cells and OCI-AML5 cells

As shown in Fig. 3c, with the addition of 4′-O-Methylbroussochalcone B, the migration of ML-2 cells and OCI-AML5 cells was evidently inhibited which was proportional to the concentration. Meanwhile, the expression level of migration related markers (N-cadherin, E-cadherin, Vimentin, Snail-1, and Slug) were detected by Western Blot. **Figure** **3**d and e showed that 4′-O-Methylbroussochalcone B up-regulated the expression of E-Cadherin in both ML-2 cells and OCI-AML5 cells, while the expression of N-Cadherin and Vimentin were down-regulated correspondingly. The upstream transcription factor, Snail-1 and Slug were also down-regulated after treatment with 4′-O-Methylbroussochalcone B. Therefore, the results demonstrated that 4′-O-Methylbroussochalcone B could hinder the ML-2 cells and OCI-AML5 cells migration in a concentration dependent manner.

### 4′-O-Methylbroussochalcone B induced apoptosis in ML-2 cells and OCI-AML5 cells

**Fig. 4:**
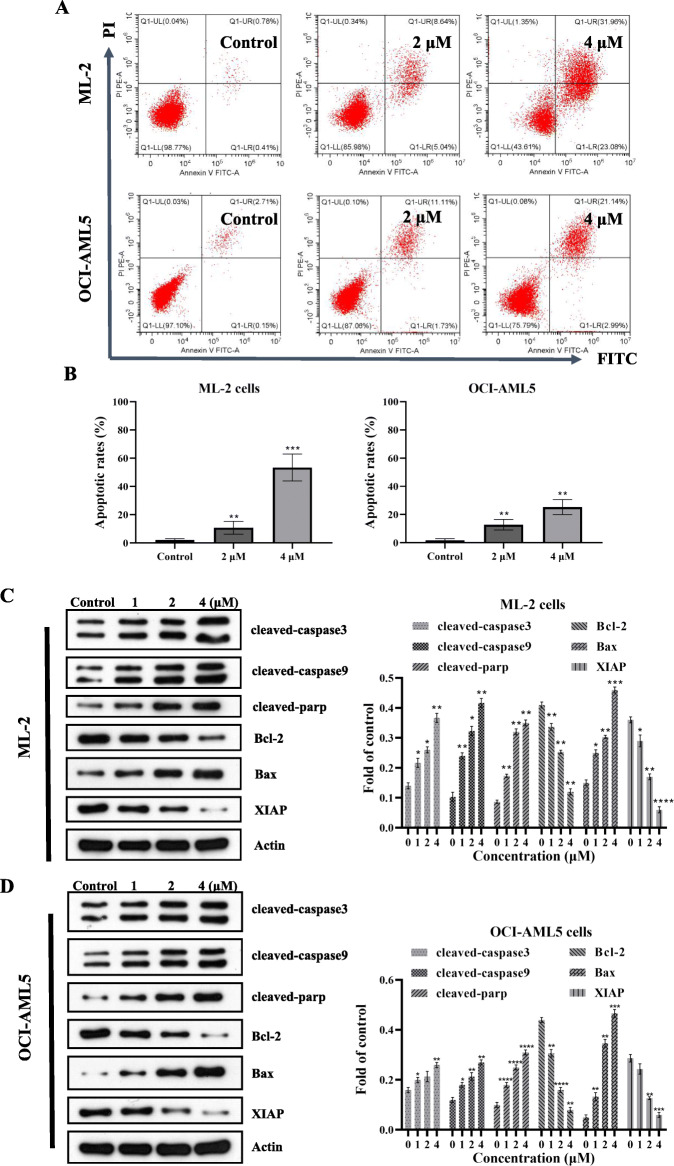
4′-O-Methylbroussochalcone B induced apoptosis and regulated apoptosis-related proteins in ML-2 cells and OCI-AML5 cells. **a**-**b** Measurement of apoptosis in ML-2 cells and OCI-AML5 cells using flow cytometry analysis; **c** Western Blot analyzed the expression level of Cleaved-caspase 3/9, cleaved-parp, Bcl-2, Bax and XIAP in ML-2 cells, full-length blots/gels are presented in Supplementary Fig. S3A; **d** Western Blot analyzed the expression level of Cleaved-caspase 3/9, cleaved-parp, Bcl-2, Bax and XIAP in OCI-AML5 cells, full-length blots/gels are presented in Supplementary Fig. S3b.The data were presented as the mean ± SEM *P < 0.05, **P < 0.01, ***P < 0.001 and ****P < 0.0001 vs the control

To explore the apoptotic effects of 4′-O-Methylbroussochalcone B on ML-2 cells and OCI-AML5 cells, we performed apoptotic analysis with Annexin V-FITC/PI double staining and quantitated by flow cytometry. As shown in Fig. 4a, b, the percentage of apoptotic ML-2 cells (including early phase and late phase apoptosis) were increased up to 13.68 and 55.04% at the indicated concentrations (2 μM and 4 μM) compared to the control. In addition, the apoptotic rates of OCI-AML5 cells with the treatment of 4′-O-Methylbroussochalcone B at 2 μM and 4 μM were 12.84 and 24.13%. The apoptotic rates of both were concentration dependent. It illustrated that 4′-O-Methylbroussochalcone B could induce the apoptosis of ML-2 cells and OCI-AML5 cells.

### 4′-O-Methylbroussochalcone B regulated apoptosis-related proteins in ML-2 cells and OCI-AML5 cells

To further explore the mechanisms of 4′-O-Methylbroussochalcone B inducing apoptosis, the expression of key proteins involved the mitochondria-related apoptosis pathway was examined by Western Blot (Fig. 4c, d). Cleaved-caspase 3/9 and cleaved-parp activation were observed after treatment with 4′-O-Methylbroussochalcone B at different concentrations (0, 1 μM, 2 μM and 4 μM) in ML-2 cells and OCI-AML5 cells. The pro-apoptosis protein, Bax, was up-regulated in a concentration-dependent manner, while the anti-apoptosis protein Bcl-2 showed an opposite trend. The expression level of X-linked inhibitor of apoptosis protein (XIAP) was decreased in ML-2 cells and OCI-AML5 cells with the treatment of 4′-O-Methylbroussochalcone B. These revealed that 4′-O-Methylbroussochalcone B could induce the apoptosis of ML-2 cells and OCI-AML5 cells by regulating apoptosis-related proteins.

### 4′-O-Methylbroussochalcone B inhibited the MAPK signaling pathway in ML-2 cells and OCI-AML5 cells

**Fig. 5:**
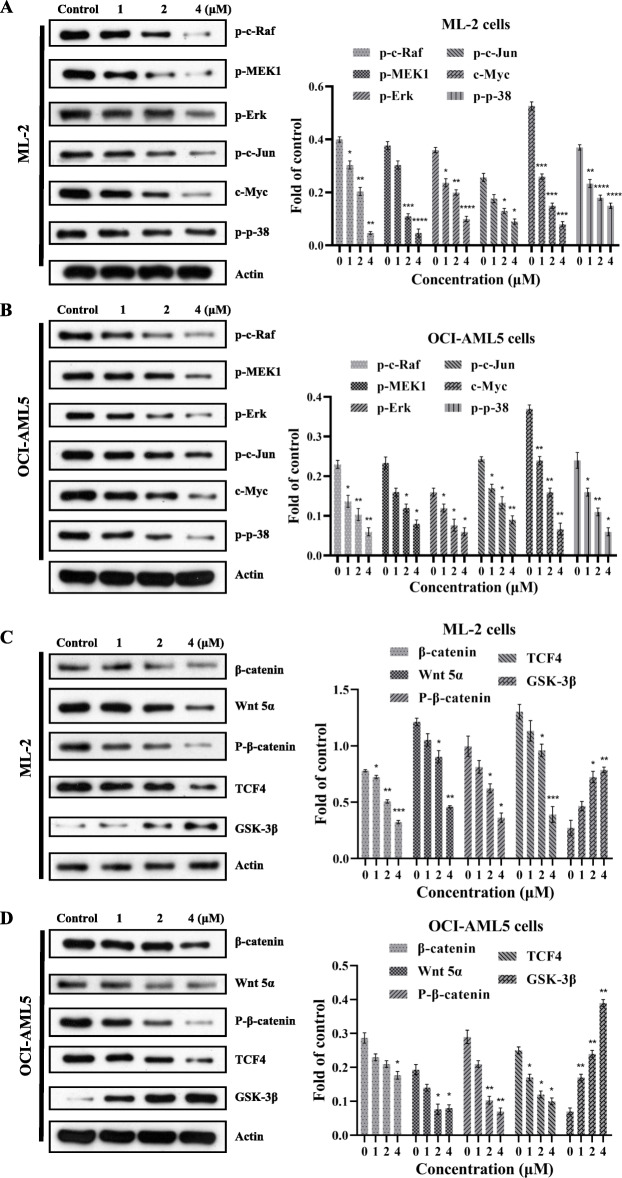
The expression level of proteins MAPK of signaling and Wnt/β-catenin pathway was determined in ML-2 cells and OCI-AML5 cells with the treatment of 4′-O-Methylbroussochalcone B. **a**-**b** Western Blot analyzed the expression level of p-c-Raf, p-MEK1, p-Erk, p-c-Jun, c-Myc and p-p-38 in ML-2 cells and OCI-AML5 cells, full-length blots/gels are presented in Supplementary Fig. S4. **c**-**d** Western Blot analyzed the expression level of β-catenin, Wnt 5α, P-β-catenin, TCF4 and GSK-3β in ML-2 cells and OCI-AML5 cells, full-length blots/gels are presented in Supplementary Fig. S5. The data were presented as the mean ± SEM *P < 0.05, **P < 0.01, ***P < 0.001 and ****P < 0.0001 vs the control

In order to investigate whether 4′-O-Methylbroussochalcone B has an effect on MAPK signaling pathway, the expression level of related MAPK signaling proteins (p-c-Raf, p-MEK1, p-c-Jun, p-Erk, c-Myc and p-p-38) were determined in ML-2 cells and OCI-AML5 cells treated with 4′-O-Methylbroussochalcone B for 48 h. As shown in **Fig.** **5****a** and **b**, the expressive protein level of p-c-Raf, p-MEK1, p-c-Jun, p-Erk, c-Myc and p-p-38 were all downregulated in a concentration-dependent manner after the treatment of 4′-O-Methylbroussochalcone B. All these results indicated that 4′-O-Methylbroussochalcone B could inhibit the activation of MAPK signaling pathway in ML-2 cells and OCI-AML5 cells.

### 4′-O-Methylbroussochalcone B inhibited the Wnt/β-catenin signaling pathway in ML-2 cells and OCI-AML5 cells

According to the inhibitory results of proliferation and migration, it was hypothesized that 4′-O-Methylbroussochalcone B treatment might affect the Wnt/β-catenin signaling pathway. To confirm this property, the expression of related Wnt/β-catenin signaling proteins (β-catenin, Wnt 5α, P-β-catenin, TCF4 and GSK-3β) were determined. As shown in **Fig.** **5****c** and **d**, 4‘-O-Methylbroussochalcone B could decrease the expression level of β-catenin, Wnt 5α, P-β-catenin and TCF4. In contrast, the expression level of GSK-3β was significantly increased with the treatment of 4’-O-Methylbroussochalcone B in ML-2 cells and OCI-AML5 cells. Hence, these findings reflected that 4′-O-Methylbroussochalcone B could hinder the Wnt/β-catenin signaling pathway in ML-2 cells and OCI-AML5 cells.

## Discussion

Human AML is characterized by abnormal proliferation of undifferentiated and non-functional hematopoietic cells in the bone marrow [17]. Improvements in supportive care have increased overall survival rates in younger patients (< 15 years of age) to more than 60%, however, 40% relapses requiring salvage therapy ultimately [18]. Extensive research have revealed that chemotherapy drugs might be an effective strategy to treat human AML [19]. *Cullen corylifolium* is a plant widely used in traditional Chinese medicine for its stomachic, anthelmintic, and diuretic properties [20]. Some natural chalcones such as Corylin from *Cullen corylifolium* have been reported as antitumor agents [21]. In the current study, six chalcones (4′-O-Methylbroussochalcone B, Broussochalcone B, Isobavachalcone, Bavachromene, Isobavachromene, and Dorsmanin A) from *Cullen corylifolium* were evaluated for their antiproliferative effects against the AML cells initially. Among all these chalcones, 4′-O-Methylbroussochalcone B displayed the best antiproliferative effects against the cells. All these antiproliferative results indicated that 4′-O-Methylbroussochalcone B have the potential to be a potently antitumor agent against acute myeloid leukemia.

Microtubules as dynamic polymers of α-tubulin and β-tubulin have been one of the best targets for developing anti-cancer drugs [22]. These microtubules targeted agents are acted through two processes (i) inhibiting depolymerization of tubulin and (ii) inhibiting polymerization of tubulin [23]. Now days, various binding domains have been explored that the three major binding domain of tubulin are taxol, vinca and colchicine binding domain [24]. Tubulin inhibitors that bind to the colchicine-binding site have potential ability to treat cancer [25, 26]. Our results demonstrated that 4′-O-Methylbroussochalcone B could be a novel tubulin polymerization inhibitor. The EBI assay in ML-2 cells indicated that 4′-O-Methylbroussochalcone B can directly bind to the cochicine binding site of β-tubulin. In addition, the molecular docking study was carried out to elucidate the binding features of 4′-O-Methylbroussochalcone B with tubulin.

Microtubules play an important role in mitosis and cell division [27]. It was reported that MD_S,_ a kind of microtubules and the derivatives of chalcones, suppressed tubulin polymerization and arrested G2/M cell-cycle [25]. Our results also showed that 4′-O-Methylbroussochalcone B caused G2/M cell-cycle in ML-2 cells and OCI-AML5 cells which was proportional to the concentration. Based on the reported references, tubulin polymerization inhibitors could induce apoptosis against different cancer cells [28]. Thus, we further explored the apoptotic effects of 4′-O-Methylbroussochalcone B against ML-2 cells and OCI-AML5 cells. From the results of apoptosis assay, 4′-O-Methylbroussochalcone B could induce apoptosis and affect the expression level of apoptosis related proteins in a concentration dependent manner in ML-2 cells and OCI-AML5 cells.

Chalcone analogues notably suppressed on the migration of HUVEC cells [29]. In this work, 4′-O-Methylbroussochalcone B inhibited the migration of ML-2 cells and OCI-AML5 cells obviously. Meanwhile, the expression level of migration related proteins were determined. The expression level of E-Cadherin was up-regulated by 4′-O-Methylbroussochalcone B, while the level of N-Cadherin and Slug was down-regulated correspondingly.

The mitogen-activated protein kinase (MAPK) cascade is a critical pathway for human cancer cell survival to drug therapy [30]. There are four independent MAPK pathways composed of four signaling families: the MAPK/ERK family, c-Jun N-terminal kinase (JNK), p38, and Big MAP kinase-1 (BMK-1) signaling families [31]. In order to explore the effects of 4′-O-Methylbroussochalcone B on MAPK pathway, the MAPK pathway related proteins (p-c-Raf, p-MEK1, p-c-Jun, p-Erk, c-Myc, and p-p-38) were investigated their expression level in ML-2 cells. Based on the Western Blot results, 4′-O-Methylbroussochalcone B down-regulated the expressive protein level of p-c-Jun, p-Erk, c-Myc, and p-p-38 in a concentration-dependent manner. In other words, in ML-2 cells and OCI-AML5 cells, 4′-O-Methylbroussochalcone B could inhibit the MAPK signaling pathway. The Wnt/β-catenin signaling pathway as an evolutionarily conserved and complex signaling cascade is associated with a host of physiological and pathophysiological processes, including embryonic patterning, cell proliferation, cell differentiation, angiogenesis, and cancer [32]. In addition, Wnt/β-catenin pathway could affect migration process in some cancer cell lines [33]. To explore the effects of Wnt/β-catenin pathway, the expression level of (β-catenin, Wnt 5α, P-β-catenin, TCF4 and GSK-3β) were determined. We found that the expression level of β-catenin, Wnt 5α and P-β-catenin were decreased, on the contrary, the expression level of GSK-3β was increased in a concentration dependent manner. In a nutshell, 4′-O-Methylbroussochalcone B inhibited Wnt/β-catenin pathway in ML-2 and OCI-AML5 cells. The role of Wnt/β-catenin pathway in the process that 4′-O-Methylbroussochalcone B inhibited migration will be studied in our next work. Based on the complexity of the etiology in leukemia and the diversity of tubulin pharmacology, we will devote ourselves to the discovery of new drugs and the research of new drug mechanisms in the future.

## Conclusion

In conclusion, 4′-O-Methylbroussochalcone B potently inhibited proliferation and migration and expedited apoptosis in AML cells. 4′-O-Methylbroussochalcone B could affect the MAPK and Wnt/b-Catenin pathways. It might be a novel tubulin polymerization inhibitor targeting colchicine site to treat AML.

## Supplementary Information

**Additional file 1: Figure S1.** Cullen corylifolium and chemical structues of natural chalcones in this study.

**Additional file 2: Figure S2.** The uncropped full-length gels and blots for (A) Fig. 2c, (B) Fig. 3d and (C) Fig. 3e in ML-2 cells and OCI-AML5 cells, respectively. Each lane was labelled according to the cropped gels/blots in Fig. 2c, Fig. 3d and Fig. 3e.

**Additional file 3: Figure S3.** The uncropped full-length gels and blots for (A) Fig. 4c and (B) Fig. 4d in ML-2 cells and OCI-AML5 cells, respectively. Each lane was labelled according to the cropped gels/blots in Fig. 4c and Fig. 4d.

**Additional file 4: Figure S4.** The uncropped full-length gels and blots for (A) Fig. 5a and (B) Fig. 5b in ML-2 cells and OCI-AML5 cells, respectively. Each lane was labelled according to the cropped gels/blots in Fig. 5a and Fig. 5b.

**Additional file 5: Figure S5.** The uncropped full-length gels and blots for (A) Fig. 5c and (B) Fig. 5d in ML-2 cells and OCI-AML5 cells, respectively. Each lane was labelled according to the cropped gels/blots in Fig. 5c and Fig. 5d.

**Additional file 6: Table S1.** Inhibition of Tubulin Polymerization

## Data Availability

The analyzed data sets generated during the study are available from the corresponding author on reasonable request.

## Abbreviations

AML: Acute myeloid leukaemia
MTT: Thiazolyl blue tetrazolium bromide
MAPK: Mitogen-activated protein kinase
EMT: Epithelial-mesenchymal transition
FBS: Fetal bovine serum
EBI *N*,*N*′: Ethylenebis(iodoacetamide)
