# Supplementary figures and images for: 4’-O-Methylbroussochalcone B as a novel tubulin polymerization inhibitor suppressed the proliferation and migration of acute myeloid leukaemia cells
Source: BMC Cancer. 2021 Jan 22;21:91. doi: 10.1186/s12885-020-07759-4 (PMC7825173; doi:10.1186/s12885-020-07759-4)

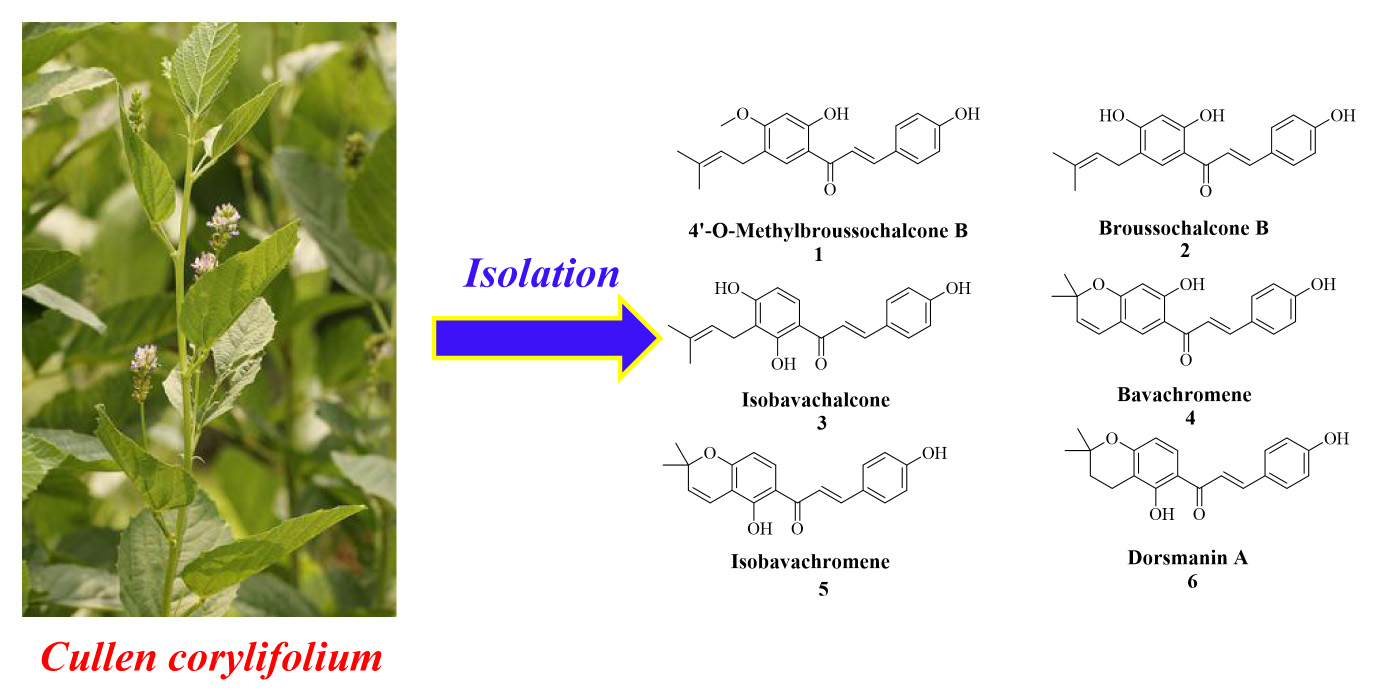

Supplement: Supplementary file 1 — Additional file 1: Figure S1. Cullen corylifolium and chemical structues of natural chalcones in this study. [file 12885_2020_7759_MOESM1_ESM.jpg]

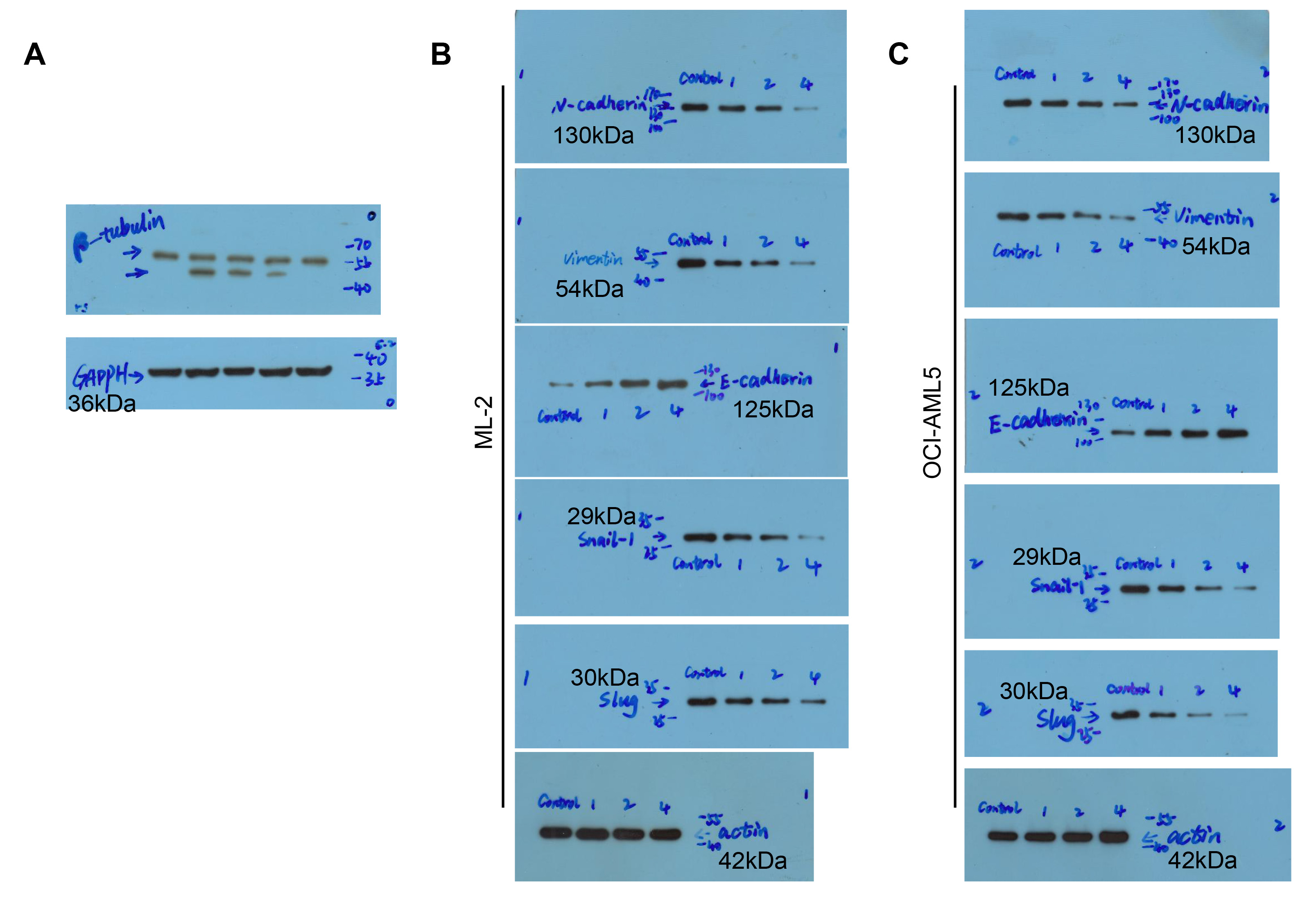

Supplement: Supplementary file 2 — Additional file 2: Figure S2. The uncropped full-length gels and blots for (A) Fig. 2c, (B) Fig. 3d and (C) Fig. 3e in ML-2 cells and OCI-AML5 cells, respectively. Each lane was labelled according to the cropped gels/blots in Fig. 2c, Fig. 3d and Fig. 3e. [file 12885_2020_7759_MOESM2_ESM.jpg]

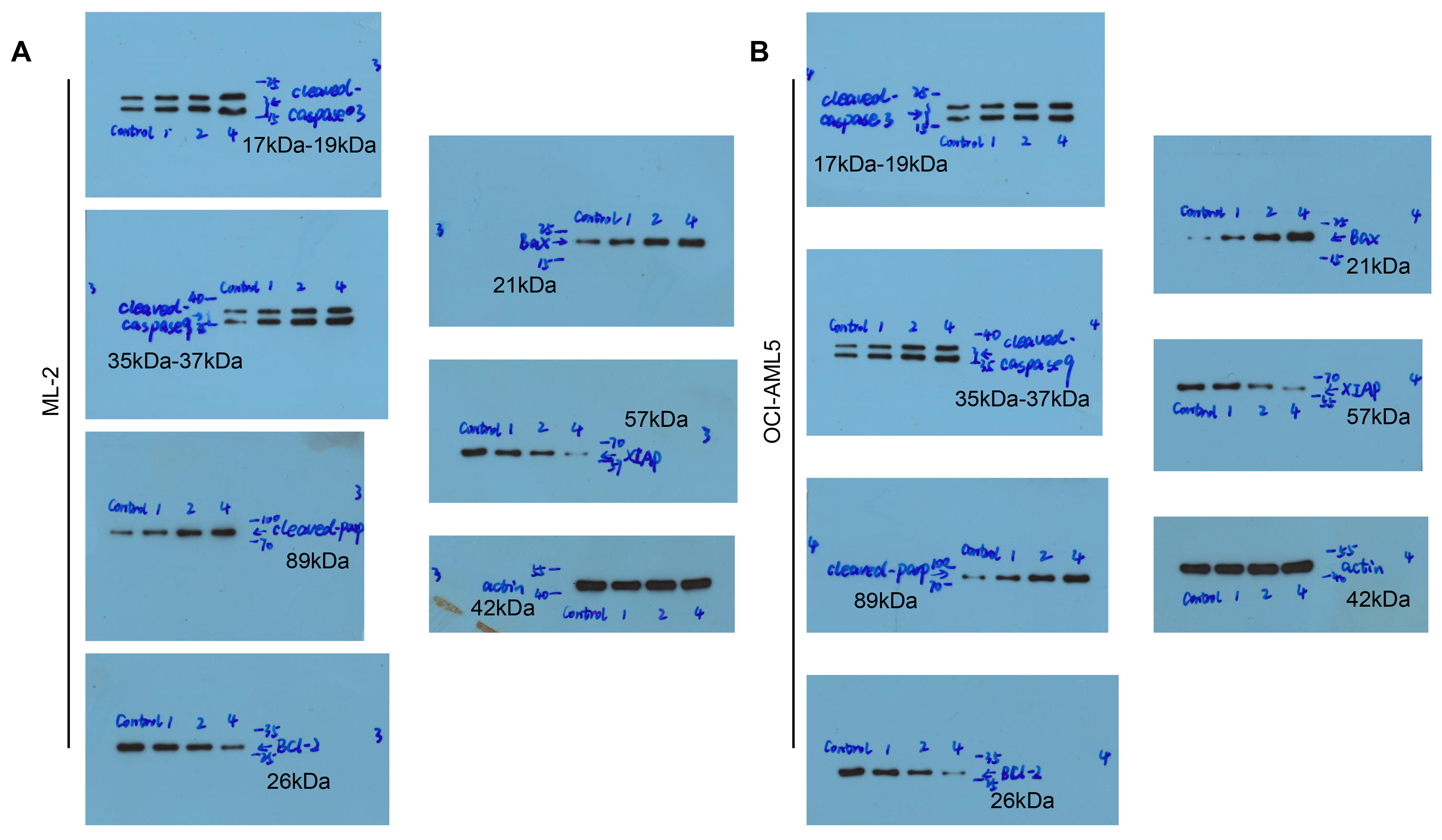

Supplement: Supplementary file 3 — Additional file 3: Figure S3. The uncropped full-length gels and blots for (A) Fig. 4c and (B) Fig. 4d in ML-2 cells and OCI-AML5 cells, respectively. Each lane was labelled according to the cropped gels/blots in Fig. 4c and Fig. 4d. [file 12885_2020_7759_MOESM3_ESM.jpg]

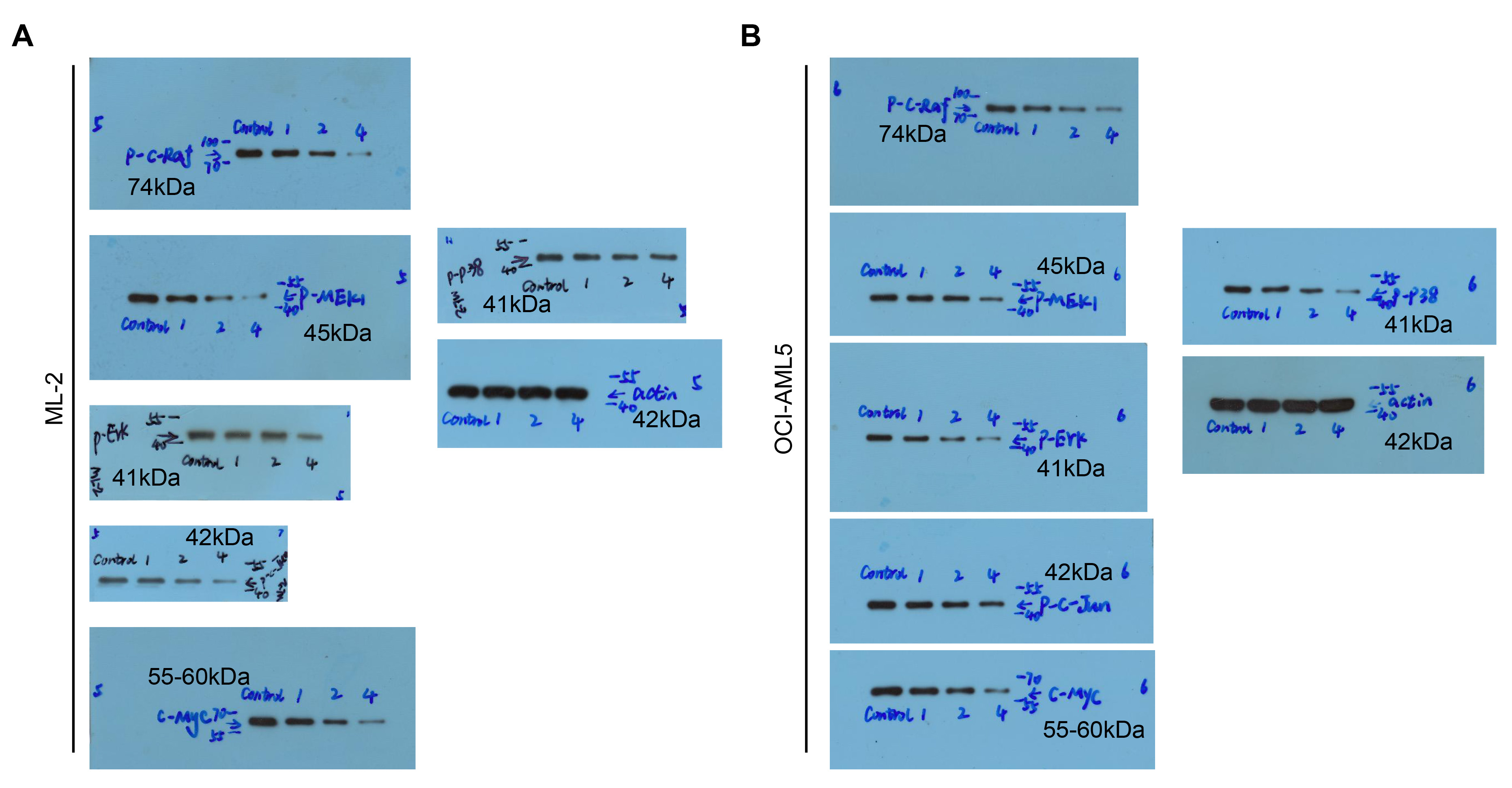

Supplement: Supplementary file 4 — Additional file 4: Figure S4. The uncropped full-length gels and blots for (A) Fig. 5a and (B) Fig. 5b in ML-2 cells and OCI-AML5 cells, respectively. Each lane was labelled according to the cropped gels/blots in Fig. 5a and Fig. 5b. [file 12885_2020_7759_MOESM4_ESM.jpg]

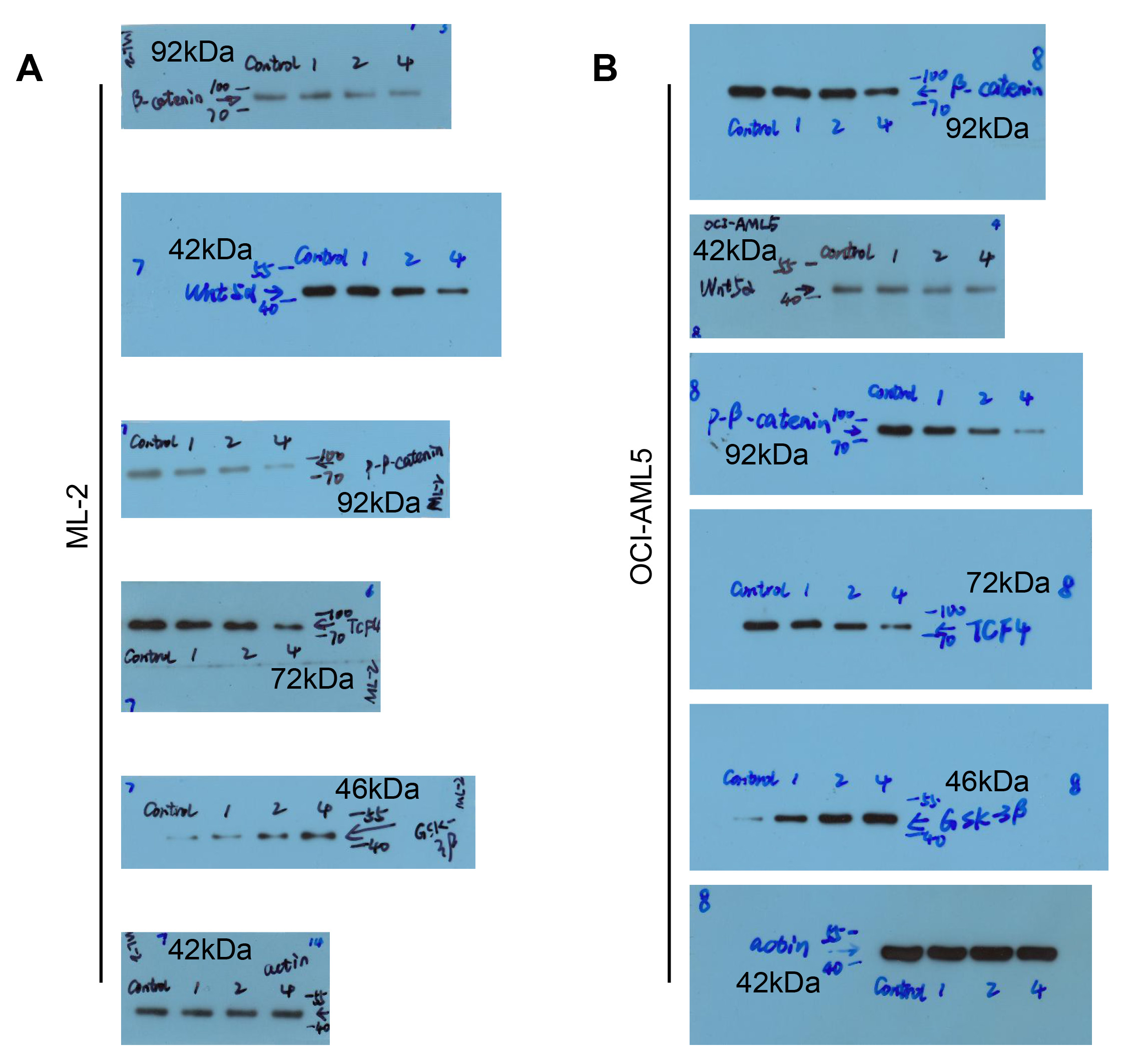

Supplement: Supplementary file 5 — Additional file 5: Figure S5. The uncropped full-length gels and blots for (A) Fig. 5c and (B) Fig. 5d in ML-2 cells and OCI-AML5 cells, respectively. Each lane was labelled according to the cropped gels/blots in Fig. 5c and Fig. 5d. [file 12885_2020_7759_MOESM5_ESM.jpg]
