# Supplementary material for: 4’-O-Methylbroussochalcone B as a novel tubulin polymerization inhibitor suppressed the proliferation and migration of acute myeloid leukaemia cells
Source: BMC Cancer. 2021 Jan 22;21:91. doi: 10.1186/s12885-020-07759-4 (PMC7825173; doi:10.1186/s12885-020-07759-4)
Supplement: Supplementary file 6 — Additional file 6: Table S1. Inhibition of Tubulin Polymerization [file 12885_2020_7759_MOESM6_ESM.docx]

# 4'-O-Methylbroussochalcone B as a novel tubulin polymerization inhibitor suppressed the proliferation and migration of acute myeloid leukaemia cells

Ziying Liu^1^, Pengfei Xu^2^, Dehua Liao^3^, Jun Zhang^4^, Changshui Wang^2^, Pei Jiang^2^*

*^1^Department of pediatrics, Affiliated Hospital of Jining Medical University, Jining Medical University, Jining, China*

*^2^Institute of Clinical Pharmacy & Pharmacology, Jining First People’s Hospital, Jining Medical University, Jining, China*

*^3^Department of Pharmacy, Hunan Cancer Hospital, Changsha, China*

*^4^Department of Pharmacy, The First Affiliated Hospital of Zhengzhou University, Zhengzhou, China*

# Correspondence to: Pei Jiang: jiangpeicsu@sina.com

**Table S1. Inhibition of Tubulin Polymerization**

| **Compound** | **IC_50_ (μM)^a^** |
| --- | --- |
| 4'-O-Methylbroussochalcone B | 2.86±0.17 |
| Broussochalcone B | 5.13±0.45 |
| Isobavachalcone | 6.38±0.12 |
| Bavachromene | 3.11±0.10 |
| Isobavachromene | 8.24±0.74 |
| Dorsmanin A | 4.39±0.26 |

The tubulin assembly assay measured the extent of assembly of 2 mg/mL tubulin after 60 minutes at 37 °C. Data are presented as mean from three independent experiments.
